# Hemotoxic Snakebite Presenting with Bilateral Blindness Due to Ischemic Occipital Infarcts

**DOI:** 10.5005/jp-journals-10071-23125

**Published:** 2019-02

**Authors:** Kodiatte Abraham A, Livingston John

**Affiliations:** 1 Department of Medicine, Christian Medical College and Hospital, Ludhiana, Punjab, India; 2 Department of Cardiology, Christian Medical College and Hospital, Ludhiana, Punjab, India

**Keywords:** Cortical blindness, Hemotoxic snakebite, Ischemic infarcts

## Abstract

Cerebrovascular complications are rare following a Viperidae snake envenomation, let alone ischemic ones. This catastrophic hemorrhaging cascade is widely known to cause a wide array of manifestations. Its manifestations can range from skin bleeds to fatal intracranial or organ hemorrhages. Our patient had cortical blindness secondary to an ischemic occipital infarct following a hemotoxic snakebite - a seemingly distinct oxymoron. The physician should be mindful of the fact that a hemotoxic snakebite can deceptively bring in ischemic attacks as well. Toxic vasculitis, thrombotic angiopathies, widespread vasospasm and endothelial damage are believed to shoulder a part of the disease process that can bring about tissue ischemia.

**Key Messages:**

Hemotoxic snake envenomation can have devastating effects. Apart from dealing with the threat of coagulopathy, the physician must also be alert to the ironical ischemic aftermath that can equally bring in misery. Our patient had one such complication-bilateral cortical blindness resulting from bilateral occipital ischemic infarcts. The physician must be aware that a hemotoxic snakebite can even instigate ischemic dilemmas, i.e. cerebrovascular infarcts, as well.

**How to cite this article:**

Kodiatte Abraham A, Livingston J. Hemotoxic Snakebite Presenting with Bilateral Blindness Due to Ischemic Occipital Infarcts. Indian J of Crit Care Med 2019;23(2): 99-101.

## INTRODUCTION

**S**nakebites and the systemic complications accompanying envenomation continues to threaten and impair quality of life through the ruthless effects of its toxins. Hemotoxic snake envenomation is very well known to trigger a devastating sequelae of events ultimately leading to a violent, untimely demise. Its management involves a swift assessment and initiation of antisnake venom (ASV) alongwith furnishing an indispensable supportive line of treatment-a daunting, but rewarding mission, with a precise modus operandi. The timely application of the 20-minute whole blood clotting time (20WBCT) can be used as a trustworthy gauge for treatment especially in a resource-restricted setting. Once the envenomation cascade commences, the morbidity and mortality escalates. Simply put, the clinical syndrome conventionally comprises of ‘bleeding organs and organ failures’. It should be recalled that although an oozing, hemorrhaging patient is an exemplar of a hemotoxic snake envenomation, it can result in ischemic infarctions as well. Our patient developed multiple brain infarctions which resulted in a poor sensorium and bilateral blindness, apart from the stereotypical skin bleeding, gastrointestinal bleeding and kidney injury. This condition of cortical blindness subsequent to ischemic infarctions of the bilateral occipital areas following a hemotoxic snake envenomation is a rarity. Table 1 lists the reported causes of blindness following snakebite^1–3^. As such, over a dozen reported cases of ischemic strokes following a hemotoxic snake bite were found in the medical literature^4–21^. Cortical blindness was seen in only 4 cases^20–23^. Apart from the vicious outcomes resulting from coagulopathy, toxic vasculitis, endothelial damage and vasospasm can also lead to significant injury as seen in our affected individual.

## CASE HISTORY

We present a case of 33-year-old farmer who was brought to the Emergency ward 3 hours after being bitten on his foot by a snake while working in his plantation. Two hours following the snakebite, he developed hematuria, bruises over the skin and bleeding from his gums. Since he nor his colleagues were able to identify the species or capture the snake, his clinical syndrome was presumably attributed to a hemotoxic Viperidae snake envenomation. Minutes prior to his arrival to our center, he began speaking irrelevantly. On arrival, he was found to be delirious, both pupils equally reacting bilaterally to light and moving all of his limbs in an erratic manner. His heart rate was 104/min, blood pressure 126/72 mm Hg and a respiratory rate of 22/min. Two distinct fang marks 1.6 cm apart which were bleeding, minimal surrounding induration and erythema were seen over his left heel. He had multiple ecchymotic patches over his body and had blood oozing from his gums.

**Table 1:** Reported causes of blindness following snakebite

• Snake venom ophthalmia (acute contact reaction of the ocular surface to venom from the spitting cobra, causing corneal opacities and ulcers or global necrosis) • Acute angle closure glaucoma following capillary leak syndrome • Vitreous hemorrhage (following hemotoxic snake envenomation) • Retinal hemorrhage, infarctions and detachments • Optic neuritis (Following neurotoxic snake envenomation) • Postneuritic optic atrophy (following neurotoxic snake envenomation) • Occipital infarction

**Table 2:** Blood investigations

*Parameter*				
	*At the time of arrival*	*6 hours after infusion of 1st batch of ASV vials*	*6 hours after infusion of 2nd batch of ASV vials*	*6 hours after infusion of 3rd batch of ASV vials*
Hemoglobin (g/dL)	11.4	11.0	10.9	10.7
Total leucocyte count (cells/mm^3^)	23,300	21,000	22,700	27,300
Platelet count (cells/mm^3^)	1,90,000	1,67,000	1,58,000	1,48,000
Prothrombin time (INR)	37.8/10.5 (3.6)	29.4/10.5 (2.8)	16.8/10.5 (1.6)	11.5/10.5 (1.1)
Activated partial thromboplastin time	40.5/28	36.5/28	29.5/28	29.5/28
Blood urea (mg/dL)	66	52	42	34
Serum creatinine (mg/dL)	1.2	1.3	1.2	1.5
Serum sodium (mEq/L)	148	138	134	138
Serum potassium (mEq/L)	3.6	4.3	4.1	3.7
Serum chloride (mEq/L)	114	115	110	107
Serum creatinine phosphokinase (mg/dL)	5950	6120	5850	5340
Fibrinogen (mg/dL)	403	386	354	412
D-dimer	Negative	Negative	Negative	Negative
20WBCT	Not coagulated	Not coagulated	Not coagulated	Coagulated

With the given clinical diagnosis, a 20WBCT was performed (and expectedly found to be deranged) and 10 vials of polyvalent ASV was given. The ASV was given 3 and a half hours after the snakebite. The protocol for ASV administration, was in accordance with his clinical stability, coagulation parameters and 20WBCT. Additionally, two batches of 10 ASV vials were given, 6 hours after the first batch, 6 hours apart, each preceded by coagulation parameter testing, until it normalised (i.e. a total of 30 polyvalent ASV vials were given). His blood investigations (Table 2) additionally revealed an acute kidney injury and a raised creatinine phosphokinase levels for which a forced alkaline diuresis was initiated.

Twelve hours into his hospital stay, his coagulation parameters normalized and his clinical condition improved excepting his mentation. For this reason, an MRI brain with angiography was performed which revealed multiple non-hemorrhagic infarcts involving his bilateral occipitotemporal and parietal regions, left frontal region and right thalamus with angiography being normal. His sensorium gradually improved in the next 24 hours, was aware and was verbally able to communicate to us regarding his bilateral complete visual deficit. A local ophthalmological, fundus and cranial nerve examination of the eye revealed a normal cornea, lens, vitreous humor, retina and optic disk, nil light perception to light and normal pupillary reflexes. An elaborate search for a local cause of blindness using direct ophthalmoscopy, slit lamp examination and intraocular pressure measurement was performed, ruling out a corneal, lens-related, vitreal, retinal or optic nerve abnormality. With all local causes ruled out, his bilateral blindness was attributed to a breach in the postchiasmatic visual pathway, specifically the geniculocalcarine pathway, as a consequence of his bilateral occipital infarcts. He was discharged from hospital and is on regular follow-up and still continues to have his visual impairment.

## DISCUSSION

Our patient developed hemorrhagic manifestations, rhabdomyolysis and ischemic occipital infarcts causing cortical blindness following a probable Viperidae snakebite (with an intact anterior visual pathway) Anton syndrome (visual anosognosia) is a rare complication of cortical blindness with denial of vision loss in patients unable to see^24,25^. The patient, however, was fully aware of his visual deficits. Although it appears contradictory that a hemotoxic snakebite induces an ischemic infarct, this undeniably demonstrates the versatility of the virulent snake toxin. The non- coagulopathic effects of snake toxin have known to be an adverse consequence through various mechanisms listed in Table 3.

**Table 3:** Mechanisms of ischemic cerebrovascular infarcts

• Disseminated intravascular coagulopathy causing vessel occlusions • Hypercoagulation due to procoagulants in the venom • Hyperviscosity caused by hypovolemia and hypotension causing vessel occlusion • Few components of venom can cause severe vascular spasm, endothelial damage, and increased vascular permeability • Toxic vasculitis • Thrombotic microangiopathy

**Table 4:** Cerebrovascular ischemic manifestations of hemotoxic snakebite

*Cerebrovascular ischemic manifestations of hemotoxic snake bite*
• Frontal temporoparieto-occipital ischemic infarct • Bilateral internal capsule ischemic infarct • Cerebellar, pontine and lacunar ischemic infarct • Cerebral ischemic infarct in the left middle cerebral artery territory • Ischemic infarcts in the territory of the left middle cerebral artery, the right posterior cerebral artery, and both superior cerebellar arteries • Bilateral posterior circulation ischemic infarct • Bilateral cerebellar and occipital ischemic infarct • Bilateral thalamic ischemic infarct

The physician must be alert to the dynamic spectrum of snake toxin induced injury. Table 4 summarises the various cerebrovascular ischemic manifestations of a hemotoxic snake bite reported by various authors ^4, 8, 15–21^.

The patient survived this ordeal because of the swift com- mencement of treatment but had unfortunately experienced a rare manifestation in the process, contributing to a significant number of disease-adjusted life years.

## CONCLUSION

Hemotoxic snake envenomation can have catastrophic con- sequences. The patient had cortical blindness resulting from ischemic occipital infarcts following a hemotoxic snakebite-an apparent paradox in itself. The physician must be aware of the non- coagulopathic complications by which hemotoxic snake venom can produce which compounds the course of the syndrome.
